# A TaqMan real-time PCR assay for the detection and quantitation of *Plasmodium knowlesi*

**DOI:** 10.1186/1475-2875-9-344

**Published:** 2010-11-30

**Authors:** Paul CS Divis, Sandra E Shokoples, Balbir Singh, Stephanie K Yanow

**Affiliations:** 1 Malaria Research Centre, University Malaysia Sarawak, Sarawak, Malaysia; 2 Provincial Laboratory for Public Health, Edmonton, Canada; 3 School of Public Health, University of Alberta, Edmonton, Canada

## Abstract

**Background:**

The misdiagnosis of *Plasmodium knowlesi *by microscopy has prompted a re-evaluation of the geographic distribution, prevalence and pathogenesis of this species using molecular diagnostic tools. In this report, a specific probe for *P. knowlesi*, that can be used in a previously described TaqMan real-time PCR assay for detection of *Plasmodium *spp., and *Plasmodium falciparum, Plasmodium vivax, Plasmodium malariae *and *Plasmodium ovale*, was designed and validated against clinical samples.

**Methods:**

A hydrolysis probe for a real-time PCR assay was designed to recognize a specific DNA sequence within the *P. knowlesi *small subunit ribosomal RNA gene. The sensitivity, linearity and specificity of the assay were determined using plasmids containing *P. knowlesi *DNA and genomic DNA of *P. falciparum, P. knowlesi, P. malariae, P. ovale *and *P. vivax *isolated from clinical samples. DNA samples of the simian malaria parasites *Plasmodium cynomolgi *and *Plasmodium inui *that can infect humans under experimental conditions were also examined together with human DNA samples.

**Results:**

Analytical sensitivity of the *P. knowlesi*-specific assay was 10 copies/μL and quantitation was linear over a range of 10-10^6 ^copies. The sensitivity of the assay is equivalent to nested PCR and *P. knowlesi *DNA was detected from all 40 clinical *P. knowlesi *specimens, including one from a patient with a parasitaemia of three parasites/μL of blood. No cross-reactivity was observed with 67 *Plasmodium *DNA samples (31 *P. falciparum*, 23 *P. vivax*, six *P. ovale*, three *P. malariae*, one *P. malariae/P. ovale*, one *P. falciparum/P. malariae, one P. inui and one P. cynomolgi) *and four samples of human DNA.

**Conclusions:**

This test demonstrated excellent sensitivity and specificity, and adds *P. knowlesi *to the repertoire of *Plasmodium *targets for the clinical diagnosis of malaria by real-time PCR assays. Furthermore, quantitation of DNA copy number provides a useful advantage over other molecular assays to investigate the correlation between levels of infection and the spectrum of disease.

## Background

The sensitivity and specificity of a diagnostic test define the extent to which a pathogen can be effectively identified in a patient specimen. For malaria, the examination of thin and thick blood smears by microscopy has been the gold standard diagnostic method for over a century. This test is simple to perform, requires only a microscope and has a sensitivity of 50 parasites/μL [1]. The parasitaemia can be quantified and the species of *Plasmodium *identified based on parasite morphology. When read by an experienced microscopist, the four major species of human malaria (*Plasmodium falciparum*, *Plasmodium vivax*, *Plasmodium ovale *and *Plasmodium malariae*) can usually be discriminated.

However, a major pitfall of microscopy was recently identified in the failure of this method to distinguish between the benign *P. malariae *species and the potentially lethal primate species *Plasmodium knowlesi *[2]. Zoonotic transmission of *P. knowlesi *from monkeys to humans was previously only observed in sporadic cases [3,4] and by blood passage from monkeys to humans in laboratory controlled experiments [5-7] but was not routinely detected by microscopic analysis of patient specimens due to morphological similarities between *P. knowlesi *and *P. malariae *[8]. As such, *P. knowlesi *was not recognized as a cause of malaria in human populations, until recently. Using molecular diagnostic tools, including DNA sequencing and newly-developed *P. knowlesi*-specific PCR primers, Singh *et al *[2] examined blood samples from 208 malaria patients in the Kapit division of Malaysian Borneo, and found that none of the cases identified as *P. malariae *by microscopy were confirmed by PCR and 120 (58%) were identified as *P. knowlesi *by PCR. These findings initiated a number of research studies into the epidemiological, clinical, ecological, and parasitological factors that determine the distribution and course of *P. knowlesi *infection. It is now recognized that human *P. knowlesi *malaria occurs in many countries in South-East Asia, causing locally-acquired malaria and infections in travelers returning from these regions [9-17]. Of significant concern, approximately 1 in 10 *P. knowlesi *infections lead to severe malaria and seven deaths have been reported from this species [18-20]. *Plasmodium knowlesi *has a 24-hour erythrocytic cycle, which is the shortest among the five species of *Plasmodium *causing human malaria, and therefore correct identification and rapid treatment are essential, particularly when the parasitaemia is high.

Given the potential for misdiagnosis of *P. knowlesi *by microscopy, alternative diagnostic tests must be employed to confirm this infection. Rapid diagnostic tests from two manufacturers have been evaluated for detection of *P. knowlesi *antigens. Cross-reactivity was observed with both *P. falciparum *and *P. vivax *antigens, precluding the use of these tests for rapid diagnosis [21]. Molecular diagnostics for detection of *P. knowlesi *include nested PCR and/or sequencing [2,22], LAMP [23], and real-time PCR [24]. In clinical diagnostic and reference laboratories, particularly those in developed countries, real-time PCR is the method of choice by providing superior sensitivity, rapid results and low risk of false positives. It is also far less laborious than nested PCR, enabling high throughput screening of patient samples. A number of real-time PCR assays have been developed for malaria diagnosis but these can only detect *P. falciparum, P. vivax, P. malariae and P. ovale *[25-31]. Only one assay has been reported for detection of *P. knowlesi *by real-time PCR [24]. However, the validation of this assay was limited to 2 reference DNA samples from infections in monkeys and no human clinical samples were tested. In the current report, a real-time PCR assay for *P. knowlesi *was developed and validated with clinical samples from 40 patients infected with *P. knowlesi*. The *P. knowlesi *assay developed uses the same reaction conditions of the real-time PCR assay described by Rougemount *et al *that screens for *Plasmodium *and identifies *P. falciparum, P. vivax, P. malariae and P. ovale *using species-specific probes [30].

## Methods

### Samples

Whole blood samples were collected from patients admitted with malaria to Kapit Hospital, Sarawak from July 2006 to February 2008 for a study aimed at determining the clinical and laboratory features of knowlesi malaria [20]. Blood samples were transported in liquid nitrogen to the Malaria Research Centre, University Malaysia Sarawak and stored in the -80°C freezer. Parasitaemia was determined by examination of thick blood films by two experienced microscopists from the Malaria Research Centre, University Malaysia Sarawak and the Sarawak Health Department. The parasitaemia was estimated by counting the number of parasites per 500 white blood cells and then calculating the parasite counts from the total number of white blood cells per μL blood for each patient. The mean of the two parasitaemia for each sample was used in the data analysis. Forty patient samples with single *P. knowlesi *infections were randomly selected for this study [20]. Of these, 26 (65%) had a parasitaemia below 500 parasites per μL blood and the rest ranged from 500 to 28,000 parasites per μL blood. Genomic DNA of *P. falciparum *(2 samples), *P. vivax *(4 samples), *P. ovale *(1 sample) and *P. malariae *(1 sample) were obtained from patients at Kapit Hospital. *Plasmodium *species in all patient samples were identified and confirmed by nested PCR assays [2]. The collection and testing of blood samples was approved by the Medical Research and Ethics Committee of the Malaysian Ministry of Health. Genomic DNA samples of *Plasmodium cynomolgi *and *Plasmodium inui *were obtained from the Biomedical Primate Research Centre, Rijswijk, the Netherlands. To determine the specificity of the *P. knowlesi *probe, an additional 57 samples (29 *P. falciparum*, 19 *P. vivax*, five *P. ovale*, two *P. malariae*, one *P. malariae/P. ovale *and one *P. falciparum/P. malariae*) from the Provincial Laboratory for Public Health in Canada were tested from patients with acute malaria using the ABI 7500 platform. These samples were tested with approval from the Health Research Ethics Board of the University of Alberta.

### Plasmid DNA

Part of the 18S rRNA gene of *P. knowlesi *KH33 (accession number AY327549) was cloned into pCR^®^-Blunt vector (Invitrogen, USA) and verified by DNA sequencing. The concentration of plasmid DNA was determined by spectrophotometer for the calculation of the DNA copy number. For standard curve analysis in real-time PCR assays, the plasmid was diluted 10-fold in nuclease free water from 10^6 ^copies to 1 copy per μL. Threshold cycle (C*t*) values in real-time PCR assays were determined by the optimum standard curve produced by the dilutions of plasmid DNA.

### DNA extraction

Whole blood samples were equilibrated at room temperature and 200 μL of blood was used for DNA extraction. Genomic DNA was extracted according to the spin-column method using the QIAamp DNA Blood Mini Kit, as per the manufacturer's instructions (Qiagen, Germany) and eluted in a total volume of 200 μL for each sample.

### Nested PCR

The identification of *Plasmodium *species was performed by nested PCR as described previously [2]. For the first round of amplification, part of the 18 S rRNA gene was amplified using primers rPLU1 and rPLU5, which generates an amplicon of approximately 1650 bp in length. The determination of *Plasmodium *species was carried out in the second nested amplification reaction for the identification of *P. falciparum *(primers rFAL1, rFAL2), *P. vivax *(rVIV1, rVIV2), *P. ovale *(rOVA1, rOVA4), *P. malariae *(rMAL1, rMAL2) and *P. knowlesi *(Pmk8, Pmkr9).

### Real-time PCR

Real-time PCR was performed using TaqMan chemistry and hydrolysis probes. Two separate reactions were performed: 1) a screening reaction for the detection of all *Plasmodium *species, and 2) a specific reaction for the detection of *P. knowlesi*. Both reactions use the same primers (Plasmo 1 and 2), but distinct probes (Plasprobe and Pk probe), were utilised for screening and *P. knowlesi *identification, respectively. DNA sequences for the primers Plasmo 1 and 2 and the *Plasmodium *screening probe, Plasprobe, were reported previously [30]. The sequence of the probe specific for *P. knowlesi*, Pk probe, is the following: 5'-CTCTCCGGAGATTAGAACTCTTAGATTGCT-3'. Both Plasprobe and Pk probe were labelled with the fluorophore FAM on the 5' end with a black hole quencher BHQ-1 on the 3' end. Primers were synthesized by Integrated DNA Technologies (Iowa, USA) and probes by Biosearch Technologies, Inc. (Novato, CA, USA). The real-time PCR reaction consisted of 200 nM of each primer, 80 nM of probe, 12.5 μL TaqMan Universal Master Mix (Applied Biosystems, USA), and 5 μL of DNA in a 25 μL volume. Reactions were performed on the Mastercycler^® ^ep *realplex *platform (Eppendorf, Germany) at the University Malaysia Sarawak, and on the ABI 7500 platform (Applied Biosystems, USA) at the Provincial Laboratory for Public Health in Canada, with the following cycling conditions: 50°C for 2 min, initial denaturation at 95°C for 10 min, and 45 cycles of 95°C for 15 sec and 60°C for 1 min. Fluorescence data was collected during the annealing/extension step at 60°C. Cycle threshold (C*t*) values were analysed either by setting the threshold 10 times the standard deviation above the noise of baseline or adjusting the standard curve to optimum. The baseline was determined manually between cycles 3 and 15.

### Statistical analyses

Data were analysed using SPSS software, version 17.0. The strengths of the linear relationship between logarithms of mean parasitaemia and C*t *values for each probe were measured using the Pearson correlation coefficient. In this study, a negative correlation between parasitaemia and the C*t *values due to the influence of parasite DNA copy numbers was hypothesized.

## Results

### Assay design

A real-time PCR assay for *P. knowlesi *was developed that is complementary to the *Plasmodium *screening assay published by Rougemont *et al *for *P. falciparum, P. vivax, P. ovale *and *P. malariae *[30]. Their assay enables rapid screening of patient specimens for *Plasmodium *and has been implemented within clinical diagnostic laboratories for confirmation of the species of *Plasmodium *[32,33]. The assay uses primers that bind to DNA sequences within the 18 S rRNA gene that are highly conserved across *Plasmodium *species. Detection with the TaqMan hydrolysis probe, Plasprobe, recognizes all species of *Plasmodium *that infect humans, including *P. knowlesi *[30,33]. In addition to the *Plasmodium *screening assay, the same primers can be used with species-specific probes that bind to a variable region within the target sequence. A *P. knowlesi*-specific probe was designed in the current study, called Pk probe, to bind specifically to a 30 base pair sequence within this variable region. Of the other human *Plasmodium *species, *P. vivax *shares the most homology with this sequence, however only 23/30 bases are predicted to bind the Pk probe. Two primate species, *P. cynomolgi *and *P. inui*, share 26/30 and 25/30 sequence matches, respectively, and were therefore included in subsequent experiments to determine the specificity of the probe. A BLAST search using the Pk probe sequence did not identify any potential cross-reactivity with other pathogens or human DNA.

### Analytical validation of the *P. knowlesi *assay

To determine the analytical sensitivity of the assay, the limit of detection was identified using a plasmid containing part of the 18 S rRNA gene from *P. knowlesi*. Ten-fold serial dilutions of this plasmid were prepared and served as the template in both the Plasprobe and Pk probe real-time PCR assays (Table 1). Based on the cycle threshold (C*t*) values, the Pk probe was more sensitive in detecting the target DNA sequence compared with the Plasprobe. This was observed in all dilutions tested. Despite this, the limit of detection was 10 copies of template per PCR reaction for both real-time assays and the efficiencies of the two PCR reactions were very similar (94% for Plasprobe and 95% for Pk probe). Both assays were linear between 10 and 10^6 ^copies of template DNA.

**Table 1:** Detection of P. knowlesi target gene in plasmid KH33 and genomic DNA

Template	Concentration	Plasprobe Ct*	Pk probe Ct*
Plasmid (copies/μL)	1 × 10^6^	23.3	20.5
	1 × 10^5^	26.7	23.6
	1 × 10^4^	30.3	27.7
	1 × 10^3^	34.1	31.0
	1 × 10^2^	37.6	34.4
	10	40.3	37.5
	1	ND‡	ND‡

gDNA†(parasites/μL blood)	4480	26.7	25.6
	480	28.5	27.4
	48	32.2	31.1
	4.8	35.8	34.7
	0.48	40.2	38.8
	0.048	42.4	39.5
	0.0048	ND‡	ND‡

*Cycle threshold.

†Genomic DNA extracted from a clinical sample with single *P. knowlesi *infection.

‡ND, not detected.

The dynamic range of the assay was determined using serial dilutions of genomic DNA extracted from a patient sample infected with *P. knowlesi *(Table 1). As observed with the plasmid DNA, the Pk probe reaction had lower C*t *values, yet the overall sensitivity of the two assays was similar. Both real-time PCR assays detected *P. knowlesi *template over a dynamic range of 5 log dilutions.

### Specificity for *P. knowlesi*

*Plasmodium knowlesi *has been largely overlooked as a human pathogen because it was identified mainly as *P. malariae *in blood films by microscopy. However, nested PCR revealed the widespread distribution of this parasite in human populations based on its ability to distinguish genetic variations between this species and other human malarias [2]. To determine the specificity of the Pk probe, a number of genomic DNA templates extracted from patients infected with other species of *Plasmodium *(Table 2) were tested. No cross-reactivity was observed for DNA samples from patients with *P. vivax, P. falciparum, P. malariae *and *P. ovale *infections. Similar negative results were obtained with the Pk probe when 57 clinical samples from malaria patients were examined at the Provincial Laboratory for Public Health, Edmonton, Canada with the ABI 7500 platform. Furthermore, genomic DNA from two other primate species of *Plasmodium*, *P. cynomolgi *and *P. inui*, that have the potential to cause zoonoses [34] were examined. Both of these species were detected using the Plasprobe, but neither was detected with the Pk probe (Table 2).

**Table 2:** Assay specificity for P. knowlesi

Sample Code	Identification by nested PCR assay	Plasprobe Ct*	Pk probe Ct*
MISC23	*P. vivax*	31.2	ND†
CDK123	*P. vivax*	30.7	ND†
CDK118	*P. vivax*	27.6	ND†
SKS358	*P. vivax*	26.7	ND†
KH116	*P. falciparum*	25.1	ND†
CDK135	*P. falciparum*	26.7	ND†
CDK67	*P. malariae*	37.0	ND†
KH352	*P. ovale*	26.5	ND†
*P. cynomolgi*	*Plasmodium-positive*	15.0	ND†
*P. inui*	*Plasmodium-positive*	13.0	ND†

*Cycle threshold.

†ND, not detected.

### Validation with clinical samples from patients infected with *P. knowlesi*

**Figure 1:**
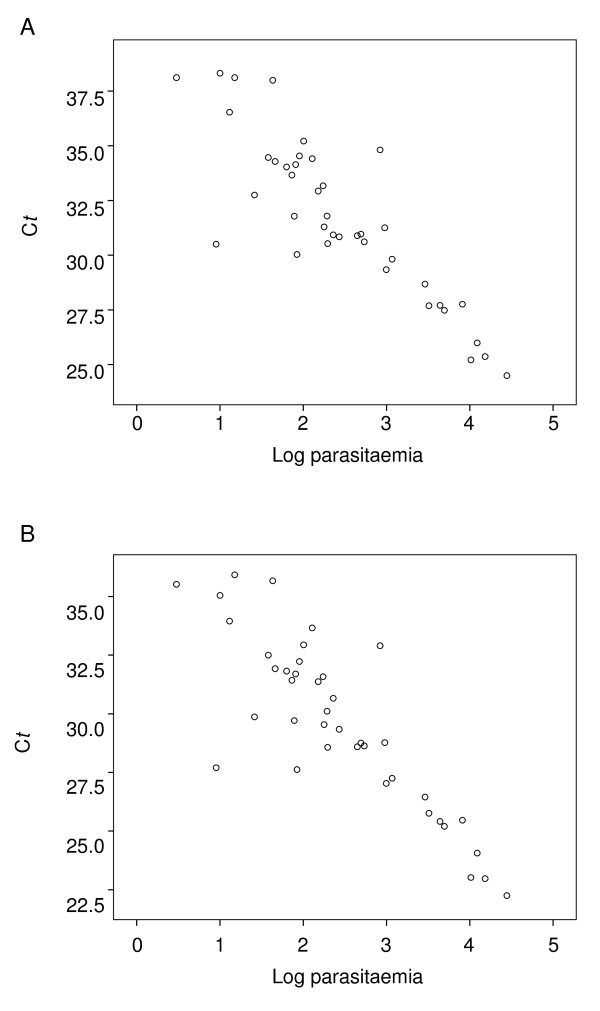
**Correlation between C*t *value and parasitaemia**. Log parasitaemia values (parasites/μL blood) were plotted against C*t *values from the Plasprobe (A) and Pk probe (B) PCR reactions, for 40 blood samples infected with *P. knowlesi*.

To evaluate the accuracy of the real-time PCR assay, 40 blood samples from patients infected with *P. knowlesi *were examined. Samples were tested alongside gDNA extracted from patients infected with other species of *Plasmodium *(described above) and five uninfected samples as negative controls. For all samples, nested PCR served as the gold standard test. The panel was tested blind. All *P. knowlesi *samples were positive with both the Plasprobe and Pk probe by real-time PCR, including a sample with a parasitaemia as low as 3 parasites/μL (Figure 1). As observed with plasmid DNA, the C*t *values were lower for the Pk probe than the Plasprobe, but the overall sensitivity was 100% for both assays. Quantitation of the template copy number by real-time PCR demonstrated increased concentrations of parasite DNA with higher parasitaemic infections. There was a significant negative correlation between C*t *values and parasitaemia (p < 0.01, Pearson correlation) for both the Plasprobe (Figure 1A) and the Pk probe (Figure 1B). Quantitative analysis of parasite DNA from clinical samples had a detection limit of 33 DNA copies/μL, corresponding to fewer than 6 parasites.

## Discussion

In this report, the validation of a real-time PCR assay with a specific probe designed to detect *P. knowlesi *is described. The analytical sensitivity of the test is 10 copies per PCR; given the multicopy nature of the rRNA gene in *Plasmodium *[35,36], this corresponds to approximately 1-2 parasite genomes. This sensitivity was corroborated with clinical samples that were detected by real-time PCR, even at a parasitaemia of 3 parasites/μL of blood.

The assay also demonstrated excellent specificity for *P. knowlesi*. No cross-reactivity was observed with other *Plasmodium *species, including two closely related primate species *P. cynomolgi *and *P. inui*. The specificity is of particular importance given the historical misdiagnosis of *P. knowlesi *by microscopy. Only with the availability of molecular methods can the prevalence of this species be confirmed in human populations. However, molecular techniques can also be subject to specificity issues. In recent studies, concerns were raised that primers used to detect *P. knowlesi *by nested PCR spuriously amplify a proportion of *P. vivax *genomic DNA samples, resulting in false positive results for *P. knowlesi *[22,37]. In the real-time PCR assay described, one set of primers amplifies all five species of *Plasmodium *but specificity is achieved through the design of the hydrolysis probe. This was supported experimentally, as no cross-reactivity of the Pk probe was observed with DNA from 65 clinical specimens infected with *Plasmodium *species other than *P. knowlesi*, including 23 *P. vivax *DNA samples. However, competition for the conserved primers may compromise the sensitivity of detection for mixed infections with *P. knowlesi*.

The *P. knowlesi *assay developed uses the same reaction conditions of a previously published real-time PCR assay by Rougemount *et al *that screens for all human species of *Plasmodium *and identifies the species using specific probes [30,33]. The real-time assay by Rougemount *et al *has been successfully implemented in two provincial public health laboratories in Canada using the ABI 7500 and 7900 platforms [32,33]. In the current study, validation for the Plasprobe and Pk probe reactions was performed on the Eppendorf platform with no loss in sensitivity compared with the ABI systems. The versatility of this test for different real-time PCR platforms is an advantage for clinical diagnostic laboratories implementing this methodology for malaria species confirmation. Other general advantages of real-time PCR include the low risk of contamination and rapid, automated processing, enabling high throughput diagnostic testing. For malaria, this test can be readily employed to evaluate patient samples that are positive for *Plasmodium *but unidentified at the species level. Samples can also be tested with the Pk probe alongside the multiplex assay for the other four species to investigate potential mixed infections [33]. Furthermore, quantitation of *P. knowlesi *DNA copy number by real-time PCR provides a measure of the level of infection, analogous to the parasitaemia calculated from a blood smear. Quantitative analysis of parasitaemia by real-time PCR can be correlated with the clinical presentation of disease to better understand the pathogenesis of this species in the human host.

## Conclusions

This study reports the analytical and clinical validation of a new real-time PCR assay for *P. knowlesi *based on TaqMan technology. The assay demonstrated excellent sensitivity, linearity and specificity with plasmid DNA and genomic DNA isolated from patients infected with *P. knowlesi*. This diagnostic tool can be useful for prospective and retrospective analysis of samples for surveillance and epidemiological studies. The impact of the under-diagnosis of *P. knowlesi *by microscopy is of global concern and rapid screening tools that can process archived samples will be invaluable to reassess the geographical distribution of this species.

## Competing interests

The authors declare that they have no competing interests.

## Authors' contributions

PCSD and SES were involved in the laboratory work (blood processing, nested PCR and real-time PCR testing), data analysis and revision of the manuscript. BS and SKY designed the experiments, provided supervision, analysis and technical assistance, and wrote the paper. All authors read and approved the final manuscript.

## References

[B1] Moody A Rapid diagnostic tests for malaria parasites Clin Microbiol Rev 2002 15 66 78 10.1128/CMR.15.1.66-78.2002 11781267 PMC118060

[B2] Singh B Kim SL Matusop A Radhakrishnan A Shamsul SS Cox-Singh J Thomas A Conway DJ A large focus of naturally acquired *Plasmodium knowlesi *infections in human beings Lancet 2004 363 1017 1024 10.1016/S0140-6736(04)15836-4 15051281

[B3] Chin W Contacos PG Coatney GR Kimball HR A naturally acquired quotidian-type malaria in man transferable to monkeys Science 1965 149 865 10.1126/science.149.3686.865 14332847

[B4] Fong YL Cadigan FC Coatney GR A presumptive case of naturally occurring *Plasmodium knowlesi *malaria in man in Malaysia Trans R Soc Trop Med Hyg 1971 65 839 840 10.1016/0035-9203(71)90103-9 5003320

[B5] Ciuca M Tomescu P Badenski G Badenski A Ionescu P Teriteanu M Contribution à l'étude de la virulence du *Plasmodium knowlesi *chez l'homme. Caratères de la maladie et biologie du parasite Arch Roumaine Path Experim Microbiol 1937 10 5 28

[B6] Garnham P Malaria parasites and other haemosporidia 1966 Oxford: Blackwell Scientific Publications

[B7] Knowles R Das Gupta B A study of monkey-malaria and its experimental transmission to man Indian Medical Gazette 1932 67 301 320 PMC5231565 29010910

[B8] Lee KS Cox-Singh J Singh B Morphological features and differential counts of *Plasmodium knowlesi *parasites in naturally acquired human infections Malar J 2009 8 73 10.1186/1475-2875-8-73 19383118 PMC2676309

[B9] Bronner U Divis PC Färnert A Singh B Swedish traveller with *Plasmodium knowlesi *malaria after visiting Malaysian Borneo Malar J 2009 8 15 10.1186/1475-2875-8-15 19146706 PMC2634766

[B10] Ennis J Teal A Habura A Madison-Antenucci S Keithly J Arguin P Barnwell J Collins W Mali S Slutsker L Dasilva A Hwang J Simian malaria in a U.S. traveler--New York, 2008 MMWR Morb Mortal Wkly Rep 2009 13 229 232 19282815

[B11] Figtree M Lee R Bain L Kennedy T Mackertich S Urban M Cheng Q Hudson BJ *Plasmodium knowlesi *in Human, Indonesian Borneo Emerg Infect Dis 2010 16 672 674 20350383 10.3201/eid1604.091624 PMC3321967

[B12] Jongwutiwes S Putaporntip C Iwasaki T Sata T Kanbara H Naturally acquired *Plasmodium knowlesi *malaria in human, Thailand Emerg Infect Dis 2004 10 2211 2213 15663864 10.3201/eid1012.040293 PMC3323387

[B13] Kantele A Marti H Felger I Muller D Jokiranta TS Monkey malaria in a European traveler returning from Malaysia Emerg Infect Dis 2008 14 1434 1436 10.3201/eid1409.080170 18760013 PMC2603100

[B14] Luchavez J Espino F Curameng P Espina R Bell D Chiodini P Nolder D Sutherland C Lee KS Singh B Human infections with *Plasmodium knowlesi*, the Philippines Emerg Infect Dis 2008 14 811 813 10.3201/eid1405.071407 18439369 PMC2600254

[B15] Ng OT Ooi EE Lee CC Lee PJ Ng LC Pei SW Tu TM Loh JP Leo YS Naturally acquired human *Plasmodium knowlesi *infection, Singapore Emerg Infect Dis 2008 14 814 816 10.3201/eid1405.070863 18439370 PMC2600232

[B16] Van den Eede P Vythilingam I Ngo DT Nguyen VH Le XH D'Alessandro U Erhart A *Plasmodium knowlesi *malaria in Vietnam: some clarifications Malar J 2010 9 20 10.1186/1475-2875-9-20 20082717 PMC2817702

[B17] Vythilingam I Noorazian YM Huat TC Jiram AI Yusri YM Azahari AH Norparina I Noorrain A Lokmanhakim S *Plasmodium knowlesi *in humans, macaques and mosquitoes in peninsular Malaysia Parasit Vectors 2008 1 26 10.1186/1756-3305-1-26 18710577 PMC2531168

[B18] Cox-Singh J Davis TM Lee KS Shamsul SS Matusop A Ratnam S Rahman HA Conway DJ Singh B *Plasmodium knowlesi *malaria in humans is widely distributed and potentially life threatening Clin Infect Dis 2008 46 165 171 10.1086/524888 18171245 PMC2533694

[B19] Cox-Singh J Hiu J Lucas SB Divis PC Zulkarnaen M Chandran P Wong KT Adem P Zaki SR Singh B Krishna S Severe malaria - a case of fatal *Plasmodium knowlesi *infection with post-mortem findings: a case report Malar J 2010 9 10 10.1186/1475-2875-9-10 20064229 PMC2818646

[B20] Daneshvar C Davis TM Cox-Singh J Rafa'ee MZ Zakaria SK Divis PC Singh B Clinical and laboratory features of human *Plasmodium knowlesi *infection Clin Infect Dis 2009 49 852 860 10.1086/605439 19635025 PMC2843824

[B21] Kawai S Hirai M Haruki K Tanabe K Chigusa Y Cross-reactivity in rapid diagnostic tests between human malaria and zoonotic simian malaria parasite *Plasmodium knowlesi *infections Parasitol Int 2009 58 300 302 10.1016/j.parint.2009.06.004 19527797

[B22] Imwong M Tanomsing N Pukrittayakamee S Day NP White NJ Snounou G Spurious amplification of a *Plasmodium vivax *small-subunit RNA gene by use of primers currently used to detect *P. knowlesi* J Clin Microbiol 2009 47 4173 4175 10.1128/JCM.00811-09 19812279 PMC2786678

[B23] Iseki H Kawai S Takahashi N Hirai M Tanabe K Yokoyama N Igarashi I Evaluation of a Loop-Mediated Isothermal Amplification (LAMP) method as a diagnostic tool of zoonotic simian malaria parasite *Plasmodium knowlesi *infection J Clin Microbiol 2010 48 2509 2514 10.1128/JCM.00331-10 20444968 PMC2897484

[B24] Babady NE Sloan LM Rosenblatt JE Pritt BS Detection of *Plasmodium knowlesi *by real-time polymerase chain reaction Am J Trop Med Hyg 2009 81 516 518 19706924

[B25] deMonbrison F Angei C Staal A Kaiser K Picot S Simultaneous identification of the four human *Plasmodium *species and quantification of *Plasmodium *DNA load in human blood by real-time polymerase chain reaction Trans R Soc Trop Med Hyg 2003 97 387 390 10.1016/S0035-9203(03)90065-4 15259463

[B26] Elsayed S Plewes K Church D Chow B Zhang K Use of molecular beacon probes for real-time PCR detection of *Plasmodium falciparum *and other *Plasmodium *species in peripheral blood specimens J Clin Microbiol 2006 44 622 624 10.1128/JCM.44.2.622-624.2006 16455928 PMC1392706

[B27] Farcas GA Zhong KJ Mazzulli T Kain KC Evaluation of the RealArt Malaria LC real-time PCR assay for malaria diagnosis J Clin Microbiol 2004 42 636 638 10.1128/JCM.42.2.636-638.2004 14766829 PMC344507

[B28] Mangold KA Manson RU Koay ES Stephens L Regner M Thomson RB Jr Peterson LR Kaul KL Real-time PCR for detection and identification of *Plasmodium *spp J Clin Microbiol 2005 43 2435 2440 10.1128/JCM.43.5.2435-2440.2005 15872277 PMC1153761

[B29] Perandin F Manca N Calderaro A Piccolo G Galati L Ricci L Medici MC Arcangeletti MC Snounou G Dettori G Chezzi C Development of a real-time PCR assay for detection of *Plasmodium falciparum*, *Plasmodium vivax*, and *Plasmodium ovale *for routine clinical diagnosis J Clin Microbiol 2004 42 1214 1219 10.1128/JCM.42.3.1214-1219.2004 15004078 PMC356834

[B30] Rougemont M Van SM Sahli R Hinrikson HP Bille J Jaton K Detection of four *Plasmodium *species in blood from humans by 18 S rRNA gene subunit-based and species-specific real-time PCR assays J Clin Microbiol 2004 42 5636 5643 10.1128/JCM.42.12.5636-5643.2004 15583293 PMC535226

[B31] Vo TK Bigot P Gazin P Sinou V De Pina JJ Huynh DC Fumoux F Parzy D Evaluation of a real-time PCR assay for malaria diagnosis in patients from Vietnam and in returned travellers Trans R Soc Trop Med Hyg 2007 101 422 428 10.1016/j.trstmh.2006.09.004 17150235

[B32] Khairnar K Martin D Lau R Ralevski F Pillai DR Multiplex real-time quantitative PCR, microscopy and rapid diagnostic immuno-chromatographic tests for the detection of *Plasmodium *spp: performance, limit of detection analysis and quality assurance Malar J 2009 8 284 10.1186/1475-2875-8-284 20003199 PMC2796674

[B33] Shokoples SE Ndao M Kowalewska-Grochowska K Yanow SK Multiplexed real-time PCR assay for discrimination of *Plasmodium *species with improved sensitivity for mixed infections J Clin Microbiol 2009 47 975 980 10.1128/JCM.01858-08 19244467 PMC2668309

[B34] Coatney GR Collins WE Warren McW Contacos PG The Primate Malarias 1972 U.S Government Printing Office, Washington D.C

[B35] Corredor V Enea V The small ribosomal subunit RNA isoforms in *Plasmodium cynomolgi* Genetics 1994 136 857 865 8005440 10.1093/genetics/136.3.857 PMC1205891

[B36] Gardner MJ Hall N Fung E White O Berriman M Hyman RW Carlton JM Pain A Nelson KE Bowman S Paulsen IT James K Eisen JA Rutherford K Salzberg SL Craig A Kyes S Chan MS Nene V Shallom SJ Suh B Peterson J Angiuoli S Pertea M Allen J Selengut J Haft D Mather MW Vaidya AB Martin DM Fairlamb AH Fraunholz MJ Roos DS Ralph SA McFadden GI Cummings LM Subramanian GM Mungall C Venter JC Carucci DJ Hoffman SL Newbold C Davis RW Fraser CM Barrell B Genome sequence of the human malaria parasite *Plasmodium falciparum* Nature 2002 419 498 511 10.1038/nature01097 12368864 PMC3836256

[B37] Sulistyaningsih E Fitri LE Loscher T Berens-Riha N Diagnostic difficulties with *Plasmodium knowlesi *infection in humans Emerg Infect Dis 2010 16 1033 1034 20507769 10.3201/eid1606.100022 PMC3086231

